# A paradoxical method to enhance compensatory lung growth: Utilizing a VEGF inhibitor

**DOI:** 10.1371/journal.pone.0208579

**Published:** 2018-12-19

**Authors:** Duy T. Dao, Lorenzo Anez-Bustillos, Sahir S. Jabbouri, Amy Pan, Hiroko Kishikawa, Paul D. Mitchell, Gillian L. Fell, Meredith A. Baker, Randolph S. Watnick, Hong Chen, Michael S. Rogers, Diane R. Bielenberg, Mark Puder

**Affiliations:** 1 Vascular Biology Program, Boston Children’s Hospital, Boston, MA, United States of America; 2 Department of Surgery, Boston Children’s Hospital, Boston, MA, United States of America; 3 Institutional Centers for Clinical and Translational Research, Boston Children’s Hospital, Boston, MA, United States of America; National Yang-Ming University, TAIWAN

## Abstract

Exogenous vascular endothelial growth factor (VEGF) accelerates compensatory lung growth (CLG) in mice after unilateral pneumonectomy. In this study, we unexpectedly discovered a method to enhance CLG with a VEGF inhibitor, soluble VEGFR1. Eight-week-old C57BL/6 male mice underwent left pneumonectomy, followed by daily intraperitoneal (ip) injection of either saline (control) or 20 μg/kg of VEGFR1-Fc. On post-operative day (POD) 4, mice underwent pulmonary function tests (PFT) and lungs were harvested for volume measurement and analyses of the VEGF signaling pathway. To investigate the role of hypoxia in mediating the effects of VEGFR1, experiments were repeated with concurrent administration of PT-2385, an inhibitor of hypoxia-induced factor (HIF)2α, via orogastric gavage at 10 mg/kg every 12 hours for 4 days. We found that VEGFR1-treated mice had increased total lung capacity (*P* = 0.006), pulmonary compliance (*P* = 0.03), and post-euthanasia lung volume (*P* = 0.049) compared to control mice. VEGFR1 treatment increased pulmonary levels of VEGF (*P* = 0.008) and VEGFR2 (*P* = 0.01). It also stimulated endothelial proliferation (*P* < 0.0001) and enhanced pulmonary surfactant production (*P* = 0.03). The addition of PT-2385 abolished the increase in lung volume and endothelial proliferation in response to VEGFR1. By paradoxically stimulating angiogenesis and enhancing lung growth, VEGFR1 could represent a new treatment strategy for neonatal lung diseases characterized by dysfunction of the HIF-VEGF pathway.

## Introduction

Vascular endothelial growth factor (VEGF) is the cardinal factor of angiogenesis [1]. While VEGF receptor 2 (VEGFR2) facilitates the key functions of VEGF, the roles of VEGFR1, or fms-like tyrosine kinase 1, are more debatable. Some evidence suggests that it serves as a decoy receptor, which exhibits regulatory function on VEGF during embryogenesis [2].

VEGF plays a critical role in pulmonary growth and development [3–5]. Dysregulation of the VEGF pathway is implicated in the pathogenesis of many pulmonary diseases of the neonate, most notably congenital diaphragmatic hernia (CDH) [6,7] and bronchopulmonary dysplasia (BPD) [8,9]. In addition, signaling from VEGFR2 is critical for physiologic processes such as compensatory lung growth (CLG) [10], and provision of VEGF accelerates alveolar regeneration after unilateral pneumonectomy (PNX) [11]. Targeting the VEGF pathway, therefore, can provide a new strategy for improving lung growth and respiratory insufficiency encountered in diseases such as CDH and BPD.

In this study, we initially aimed to inhibit CLG by using a commercially available VEGF inhibitor, VEGFR1. Soluble VEGFR1, which sequesters VEGF and prevents its activation of VEGFR2, has commonly been used to inhibit VEGF in angiogenesis research [12]. However, systemic administration of VEGFR1 at a certain concentration resulted in a paradoxical increase in lung volume. We then aimed to further characterize the physiologic effects of VEGFR1 treatment and the mechanism by which this VEGF inhibitor paradoxically enhanced CLG in the context of hypoxia-induced factor (HIF) regulation.

## Methods

### Pharmacokinetics of VEGFR1-Fc

VEGFR1 used in this study is commercially available (R&D Systems, Minneapolis, MN). It is a recombinant peptide that includes mouse VEGFR1 (Ser27-Glu759) conjugated with the Fc portion of human IgG_1_ (Pro100-Lys330). In order to assess the half-life (t_1/2_) of VEGFR1-Fc, the agent was diluted in normal saline and administered to 3 eight-week-old C57BL/6 male mice (Jackson Laboratories, Bar Harbor, ME) via intraperitoneal (ip) injection at a dose of 20 μg/kg. Fifty μL of blood was collected in ethylenediaminetetraacetic acid (EDTA)-containing tubes via retro-orbital puncture at 6, 24, 48, and 96 hours after injection. Plasma was separated by centrifugation at 4 ^o^C and 2000 g for 15 minutes. Detection of VEGFR1-Fc in plasma was achieved with a human IgG Fc enzyme-linked immunosorbent assay (ELISA) (Lifespan Biosciences, Seattle, WA) according to the manufacturer’s protocol. The half-life was derived using a one phase exponential decay model (Graphpad Prism v.7, GraphPad Software, La Jolla, CA).

### Surgical model

All procedures were carried out according to the National Institutes of Health Guide for the Care and Use of Laboratory Animals and approved by the Institutional Animal Care and Use Committee at Boston Children’s Hospital. Eight-week-old C57BL/6 male mice were anesthetized with ketamine (80–100 mg/kg) and xylazine (10–12.5 mg/kg) via ip injection. They were subsequently intubated and ventilated via a rodent ventilator (HSE-HA Minivent, Harvard Apparatus, Holliston, MA) at 150 breaths/minute. A left PNX was performed as previously described [13]. Buprenorphine was administered post-operatively every 12 hours via subcutaneous injection for 72 hours.

### Dose-response study

Mice underwent left PNX as previously described, followed by daily ip injection of VEGFR1-Fc at various doses: 0, 5, 20, 60, and 180 μg/kg. Mice were euthanized on post-operative day (POD) 4 for lung volume measurement. After euthanasia with carbon dioxide, the trachea was exposed and intubated with a 20-gauge hollow needle. The remaining right lung was removed *en bloc* with the tracheobronchial tree and inflated with 10% formalin at 30 cmH_2_O. Lung volume was measured by the water displacement method [14] and normalized for body weight.

### Repetition of the 20 μg/kg dose and pulmonary function measurement

The control (saline) and VEGFR1 (20 μg/kg) groups were repeated two more times and data were pooled from the three repetitions. On POD 4, mice were deeply anesthetized with ketamine and xylazine as previously described. Tracheotomy was again achieved with a 20-gauge needle, which was then connected to a Flexivent system (SCIREQ, Montreal, Canada). Total lung capacity and pulmonary compliance were derived from the pressure-volume loops. Total lung capacity was normalized for the animal’s weight. Mice were euthanized with carbon dioxide upon disconnection from the Flexivent machine and lung volume was measured as previously described. Lung volume was measured on POD 4 and 10. Inflated lung specimens were fixed in 10% formalin at 4 ^o^C for 24 hours before being transferred to 70% ethanol for paraffin embedding.

### Immunoblot and angiogenesis antibody array of lung tissue

Fresh lung tissue samples harvested on POD 4 from the control and VEGFR1 groups were homogenized in radioimmunoprecipitation assay (RIPA) buffer containing protease and phosphatase inhibitors (Thermo Fisher Scientific, Waltham, MA). The proteins were separated on a polyacrylamide gel, followed by transfer to a nitrocellulose membrane and blocking in TBST solution containing 5% non-fat dry milk for 1 hour. Incubation in primary antibody, which included anti-VEGFR2 (Cell Signaling Technology, Danvers, MA), -VEGF_120/164_ (R&D Systems, Minneapolis, MN), -HIF1α (Bioss, Woburn, MA), -HIF2α (Novus Biologicals, Littleton, CO), and -β-Actin (Sigma-Aldrich, St. Louis, MO), was done overnight at 4 ^o^C. Following washing with TBST for 10 minutes x3, the membrane was incubated for 1 hour at room temperature in secondary antibodies, horseradish peroxidase (HRP)-conjugated goat anti-rabbit, anti-mouse (Santa Cruz Biotechnology, Dallas, TX), or anti-rat IgG (R&D Systems, Minneapolis, MN) antibodies. The membrane was again washed and visualized with the enhanced chemiluminescence (ECL) reagents (Thermo Fisher Scientific, Waltham, MA).

Protein homogenate of one representative sample from each of the control and VEGFR1 groups was analyzed with a Proteome Profiler Angiogenesis Array (R&D Systems, Minneapolis, MN) according to the manufacturer’s protocol. Quantification of the signals were performed with ImageJ (NIH, Bethesda, MD).

### ELISA of lung, plasma, liver, and kidney

Lung, blood, liver, and kidney samples were collected on POD 4 for mice in the control and VEGFR1 groups. Blood was removed via inferior vena cava puncture and placed in collection tubes containing EDTA and plasma was separated as previously described. Right lungs, livers and left kidneys were removed and snap frozen in liquid nitrogen. At the time of analysis, protein extraction of lung, liver, and kidney samples was performed with RIPA buffer as previously described. Total protein concentration was quantified with a Bradford colorimetric assay (Bio-Rad Laboratories Inc, Hercules, CA). VEGF levels in lung, plasma, liver, and kidney as well as lung EGF levels were determined with ELISA (R&D Systems, Minneapolis, MN) according to the manufacturer’s protocol. The VEGF ELISA assay detects both VEGF_164_ and VEGF_120_ isoforms of rodent VEGF-A. EGF and VEGF concentrations of lung, liver, and kidney samples were normalized against total protein concentration.

### Quantitative Polymerase Chain Reactions (qPCR)

RNA was extracted from snap-frozen lung tissues of the control and VEGFR1 groups with TRIzol reagent (Thermo Fisher Scientific, Waltham, MA). Following reverse transcription (qScriptTM XLT cDNA SuperMix, Quanta Biosciences, Beverly, MA), amplification reactions were performed with SybrGreen reagent (Quanta Biosciences, Beverly, MA) in a StepOne Real-Time PCR System (Applied Biosystems, Foster City, CA). GAPDH was used as the housekeeping gene and relative mRNA levels were calculated using the 2^ΔΔCt^ method [15]. Target genes included surfactant proteins A-D (*Sftpa1*, *Sftpb*, *Sftpc*, and *Sftpd*) and HIF-related factors (*Hif1a*, *Hif2a*, *Vegfr1*, *Vegfr2*, *Vegfa*, *Pdk*, *Hk*, *Pdgfa*, *Pdgfb*, *Epo*, *Cxcr4*, *Cxcl12*, *Vhl*, and *Egln1*).

### Immunohistochemistry (IHC)

Formalin-fixed, paraffin-embedded lung sections were deparaffinized with xylene and progressively rehydrated in various concentrations of ethanol. Antigen retrieval was achieved with a citrate-based unmasking solution in a pressurized chamber (Decloaking Chamber, Biocare Medical, Pacheco, CA) by heating to 120 ^o^C. Slides were washed with PBST, phosphate-buffered saline containing 0.5% Triton-X, for 10 minutes x3, and blocked for 30 minutes at room temperature in PBST solution containing 1% bovine-serum albumin. Incubation with primary antibodies, goat anti-HIF2α (R&D Systems, Minneapolis, MN), rat anti-VEGF_120/164_ (R&D Systems, Minneapolis, MN), rabbit anti-Aquaporin-5 (Abcam, Cambridge, MA), rabbit anti-Vimentin (R&D Systems, Minneapolis, MN), and rabbit anti-SP-C (Abcam, Cambridge, MA), was performed overnight at 4 ^o^C. After washing with PBST for 30 minutes x3, slides were incubated in secondary antibodies, Alexa Fluor-conjugated donkey anti-rat (Abcam, Cambridge, MA), anti-rabbit, and anti-goat (Invitrogen, Carlsbad, CA) IgG antibodies. Slides were washed again with PBST, dried, and mounted.

### Inhibition of HIF2α after Left PNX

#### Experimental groups

Inhibition of HIF2α was achieved with PT-2385 (MedChemExpress, Monmouth Junction, NJ), a small-molecule inhibitor that can be administered via orogastric gavage [16]. PT-2385 was suspended in a vehicle solution comprised of 0.5% methylcellulose and 0.5% Tween 80 in water to yield a final concentration of 10 mg/mL. Mice underwent pneumonectomy as previously described and were randomized into one of the four experimental groups. The V group received gavage of the vehicle solution every 12 hours and daily administration of normal saline via ip injection. The V+R1 group received vehicle gavage and daily ip injection of VEGFR1-Fc at 20 μg/kg. The I group received PT-2385 gavage at 10 mg/kg every 12 hours and daily saline injection while the I+R1 group received both PT-2385 gavage and VEGFR1-Fc at 20 μg/kg. The dose, frequency, and route of administration of PT-2385 were chosen according to the original study investigating its effects on renal cell carcinoma [16]. Lung was harvested on POD 4 and volume was measured with the water displacement method as previously described.

#### Physical activity measurements

Before euthanasia on POD 4, mice in the four experimental groups underwent physical activity assessment. They were placed in a 42 x 42 cm open field where their rest time, walking distance, and movements were quantified for 6 minutes with an infrared-based motion tracking system (Motormonitor, Kinderscientific, Poway, CA). Movements were further classified into fine and basic movements. Fine movements refer to activities performed while standing in one place, e.g. grooming or head movements, while basic movements include instances when the animal moved from one spot to another. The experiment was carried out in 2 sets, I vs I+R1 and V vs V+R1.

#### IHC

IHC was performed as previously described with rat anti-Ki67 (Invitrogen, Carlsbad, CA) and rabbit anti-ERG (Abcam, Cambridge, MA) as primary antibodies. ERG is a nuclear marker for endothelial cells [17] and was used to facilitate counting. Slides were examined under a confocal microscope (LSM 800, Zeiss, Jena, Germany). Analysis of endothelial cell proliferation was performed at 20X magnification (N = 5 for each group). Four random fields were sampled across the entire right lung. Endothelial cells, marked by ERG, were quantified with ImageJ and proliferating endothelial cells, marked by double staining with ERG and Ki67, were counted manually. Percent proliferating endothelial cells was calculated by dividing the number of proliferating endothelial cells by total endothelial cells.

### Statistical analyses

Lung volume adjusted for body weight was compared among different doses of VEGFR1 with one-way analysis of variance (ANOVA). Comparison of lung volume between the control and VEGFR1 (20 μg/kg) groups on POD 4 and 10 was performed with two-way ANOVA. Tests of significance for pulmonary function tests, qPCR, ELISA, and immunoblot between the control and VEGFR1 groups were performed with two-sided Student’s *t*-test. Comparison of lung volume and percent proliferating endothelial cells among the four experimental groups in the HIF2α inhibition experiment was performed with ANOVA. Physical activity comparison was made by one-way ANOVA. A *P* value of < 0.05 was considered statistically significant, and all pairwise tests of significance from ANOVA models were corrected for multiple comparisons by Tukey-Kramer adjustment. Results are presented as mean ± standard error (SE). All analyses were performed with GraphPad Prism v.7 (GraphPad Software, La Jolla, CA).

## Results

### VEGFR1 paradoxically enhanced CLG at the dose of 20 μg/kg

**Fig 1:**
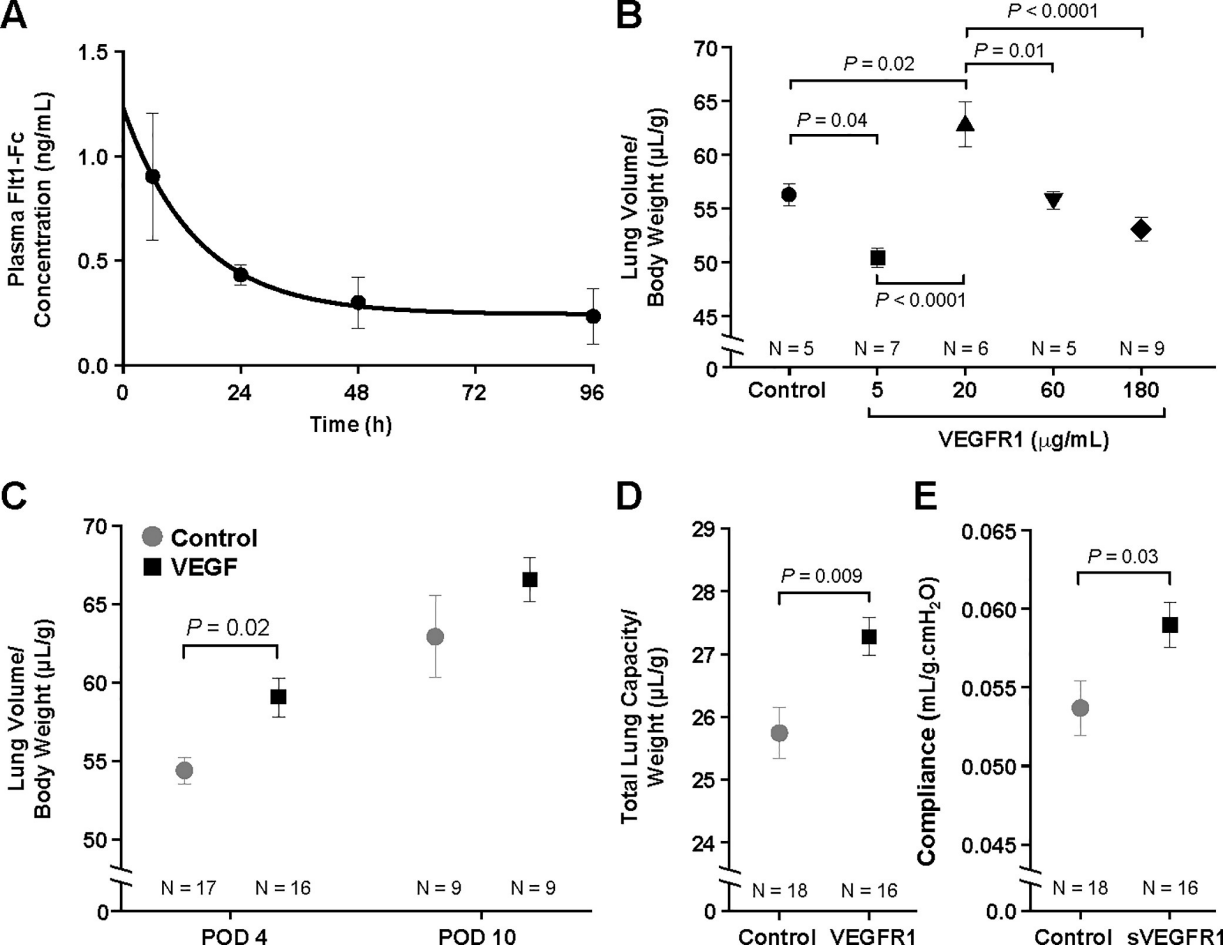
VEGFR1 paradoxically enhanced compensatory lung growth (CLG). Plasma concentration of VEGFR1-Fc was measured at various time points after injection and its half-life was measured to be 10.3 hours (A). In the dose-response study, VEGFR1 at 5 μg/kg decreased lung volume measured on post-operative day (POD) 4 after left pneumonectomy (PNX) compared to the control group (B). However, the dose of 20 μg/kg resulted in a paradoxical increase in lung growth. This result was confirmed across 3 different repetitions, where VEGFR1 at 20 μg/kg significantly improved CLG on POD 4 (C). VEGFR1 treatment also resulted in improved total lung capacity (D) and pulmonary compliance (E) on POD 4. Data are expressed as mean ± standard error (SE).

The t_1/2_ of VEGFR1-Fc was calculated to be 10.3 hours (Fig 1A). VEGFR1 was therefore administered daily via ip injection for the dose-response study. At the low dose of 5 μg/kg, VEGFR1 decreased lung volume on POD 4 as compared to the control group (50.4 ± 1.2 vs. 56.3 ± 1.5 μL/g, *P* = 0.04) (Fig 1B). However, VEGFR1 at the medium dose of 20 μg/kg resulted in a paradoxical increase in lung volume (62.9 ± 1.3 vs 56.3 ± 1.5 μL/g, *P* = 0.02). This paradoxical improvement of CLG with VEGFR1 at 20 μg/kg remained true on POD 4 when the lung volume data were pooled from 3 different repetitions under the same experimental condition (59.1 ± 1.3 vs 54.4 ± 1.2 μL/g, *P* = 0.049) (Fig 1C). In contrast, this difference no longer existed on POD 10, the end-point of CLG [18]. VEGFR1 treatment also resulted in increased total lung capacity (27.3 ±0.3 vs 25.7 ± 0.4 μL/g, *P* = 0.006) (Fig 1D) and pulmonary compliance (59.0±1.4 vs 53.7 ± 1.7 μL/cmH_2_O, *P* = 0.03) on POD 4 (Fig 1E).

In contrast to our initial hypothesis, VEGFR1 displayed various effects on CLG when administered at different doses. Specifically, the dose of 20 μg/kg resulted in a paradoxical improvement in CLG, as evidenced by both lung volume and pulmonary function measurements.

### VEGFR1 paradoxically enhanced pulmonary angiogenesis after left PNX

**Fig 2:**
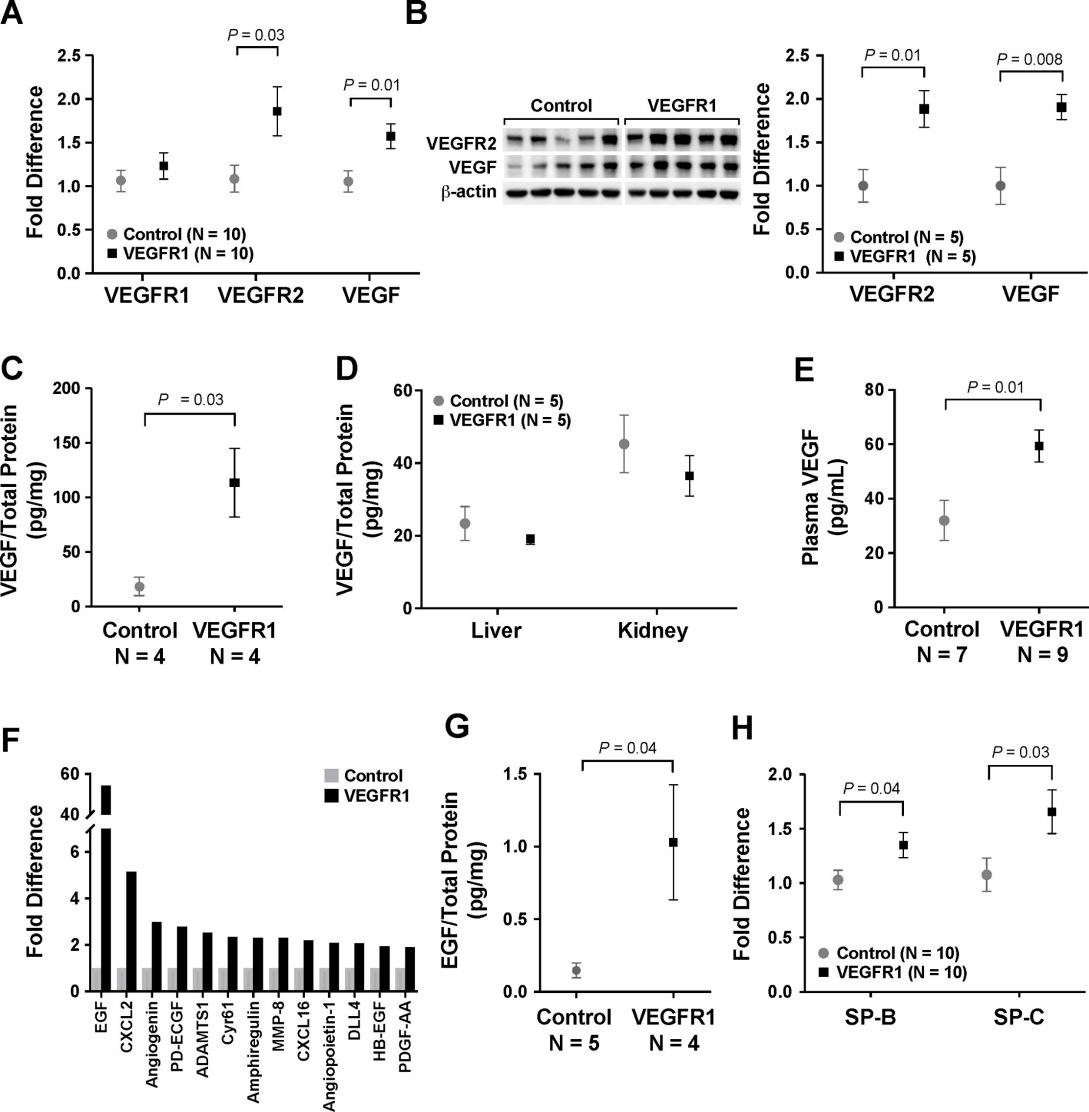
VEGFR1 increased expression of angiogenic factors and surfactant production. On quantitative polymerase chain reaction (qPCR), mice treated with VEGFR1 (20 μg/kg) demonstrated increased expression of VEGF and VEGFR2 in the lung (A). The increase in pulmonary VEGF and VEGFR2 expression was also seen on immunoblot (B). Increased pulmonary levels of VEGF with VEGFR1 treatment was also confirmed on an enzyme-linked immunosorbent assay (ELISA) (C). Meanwhile, there was no change in VEGF levels in non-regenerating organs, including liver and kidney (D). However, there was an increase in plasma VEGF concentration (E). There was also an increase in pulmonary expression of many other angiogenic and mitogenic factors, most notably epidermal growth factor (EGF), as demonstrated by an angiogenesis antibody array (F). This increase in EGF level was also confirmed by ELISA (G). VEGFR1 treatment also upregulated surfactant protein (SP)-B and -C (H). Data are expressed as mean ± standard error (SE).

Compared to the control group, lung samples from the VEGFR1 group demonstrated increased mRNA transcript levels of both VEGFR2 and VEGF on qPCR (*P* = 0.03 and 0.01, respectively) (Fig 2A). There was no difference in the expression of endogenous VEGFR1. The increase in pulmonary levels of VEGFR2 and VEGF was also confirmed on WB (*P* = 0.01 and 0.008, respectively) (Fig 2B). On ELISA, VEGF expression in the VEGFR1 group was also significantly higher (113.6 ± 31.6 vs 18.6 ± 8.5 pg/mg, *P* = 0.03) (Fig 2C). In addition, VEGF levels were measured in other organs at the same time point. While the VEGF levels in other non-regenerating organs, i.e. liver and kidney, showed no differences (Fig 2D), plasma concentration of VEGF in the VEGFR1 group was significantly higher (*P* = 0.01) (Fig 2E).

To confirm that angiogenesis was increased with VEGFR1 treatment, an antibody array was utilized to compare the expression of 53 different angiogenic factors. Of the factors that showed at least a 2-fold increase, EGF showed the strongest upregulation (Fig 2F). This result was confirmed with an EGF ELISA (*P* = 0.04) (Fig 2G). An increase in EGF expression has also been demonstrated with exogenous VEGF administration after left PNX [11]. Given that VEGFR1 increased endogenous production of VEGF, this concurrent upregulation of EGF is consistent with our previously published work. As EGF is a major mitogen for epithelial cells, the next step was to determine if VEGFR1 treatment affected the proliferation and differentiation of lung epithelial cells by quantifying the status of surfactant production, which is produced by alveolar epithelial cells (AEC) type II. Lungs from the VEGFR1 group displayed increased expression of surfactant protein B and C (SP-B and -C) (P = 0.04 and 0.03, respectively) (Fig 2H).

Collectively, these results demonstrated that VEGFR1 treatment both stimulated pulmonary angiogenesis and induced lung maturation.

### VEGFR1 treatment amplified the hypoxic response after left PNX

**Fig 3:**
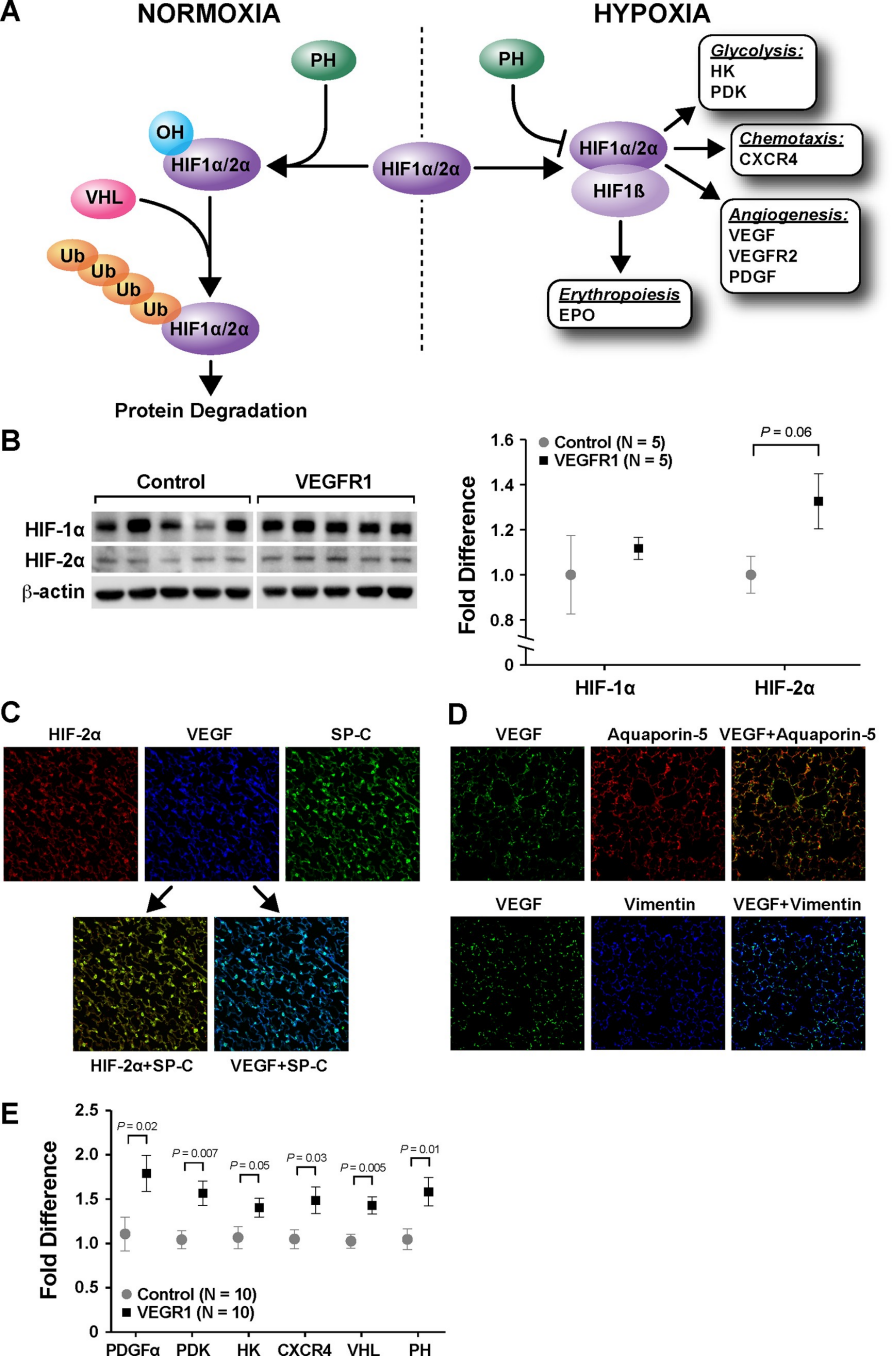
VEGFR1 treatment activated the hypoxic response after left PNX. Regulation of hypoxia-induced factor (HIF) is demonstrated (A). In a normoxic condition, HIF is hydroxylated by prolyl hydroxylase (PH), ubiquitinated by von-Hippel Lindau (VHL), and destined for degradation. In a hypoxic condition, HIF is stabilized, which leads to dimerization with HIF1β and activation of multiple signaling pathways. VEGFR1 treatment increased the expression of HIF2α in the lung (B). On immunohistochemistry (IHC), both VEGF and HIF2α colocalized with SP-C (C), indicating that they were produced in alveolar epithelial cell (AEC) type II. Furthermore, VEGF did not colocalize with Aquaporin-5, a marker for AEC type I, or Vimentin, a marker for fibroblasts (D). On qPCR, VEGFR1-treated lung also displayed increased expression of many HIF-regulated factors, including platelet-derived growth factor α (PDGFα), pyruvate dehydrogenase kinase (PDK), hexose kinase (HK), and CXC chemokine receptor 4 (CXCR4), as well as factors involved in HIF degradation, including VHL and PH (E). Data are expressed as mean ± standard error (SE).

To better understand the mechanism by which VEGFR1 upregulated the expression of VEGF and VEGFR2, pulmonary levels of HIF, a critical transcription factor that controls the expression of VEGF (Fig 3A), were measured. No significant changes in HIF1α were observed. Although not reaching statistical significance (*P* = 0.06), there was an increase in expression of HIF2α on immunoblot with VEGFR1 treatment (Fig 3B). Of note, both VEGF and HIF2α colocalized with SP-C on IHC (Fig 3C), indicating that AEC type II is the primary location in which hypoxia-stimulated production of VEGF occurs after PNX. This was further supported by the observation that VEGF did not colocalize with Aquaporin-5, a marker for AEC type I, or Vimentin, a marker for fibroblasts (Fig 3D).

Next, qPCR was used to determine the levels of other factors that are also under the control of HIF. Lung tissues from the VEGFR1 group displayed an increase in expression of many hypoxia-dependent factors, including angiogenic factor platelet-derived growth factor α (PDGFα) (*P* = 0.02), glycolytic enzymes pyruvate dehydrogenase kinase (PDK) (*P* = 0.007) and hexose kinase (HK) (*P* = 0.05), and CXC chemokine receptor 4 (CXCR4) (*P* = 0.03) (Fig 3E). In addition, there was an upregulation of proteins that are involved in the breakdown of HIF, including von Hippel-Lindau (VHL) (*P* = 0.005) and prolyl hydroxylase (PH) (*P* = 0.01).

These results suggest that VEGFR1, possibly due to its sequestration of VEGF, stimulated the hypoxic response and resulted in a paradoxical improvement in CLG. This process most likely originates from AECII, the primary site of production of HIF2α in the lung after PNX.

### Inhibition of HIF2α neutralized the effect of VEGFR1 on CLG

**Fig 4:**
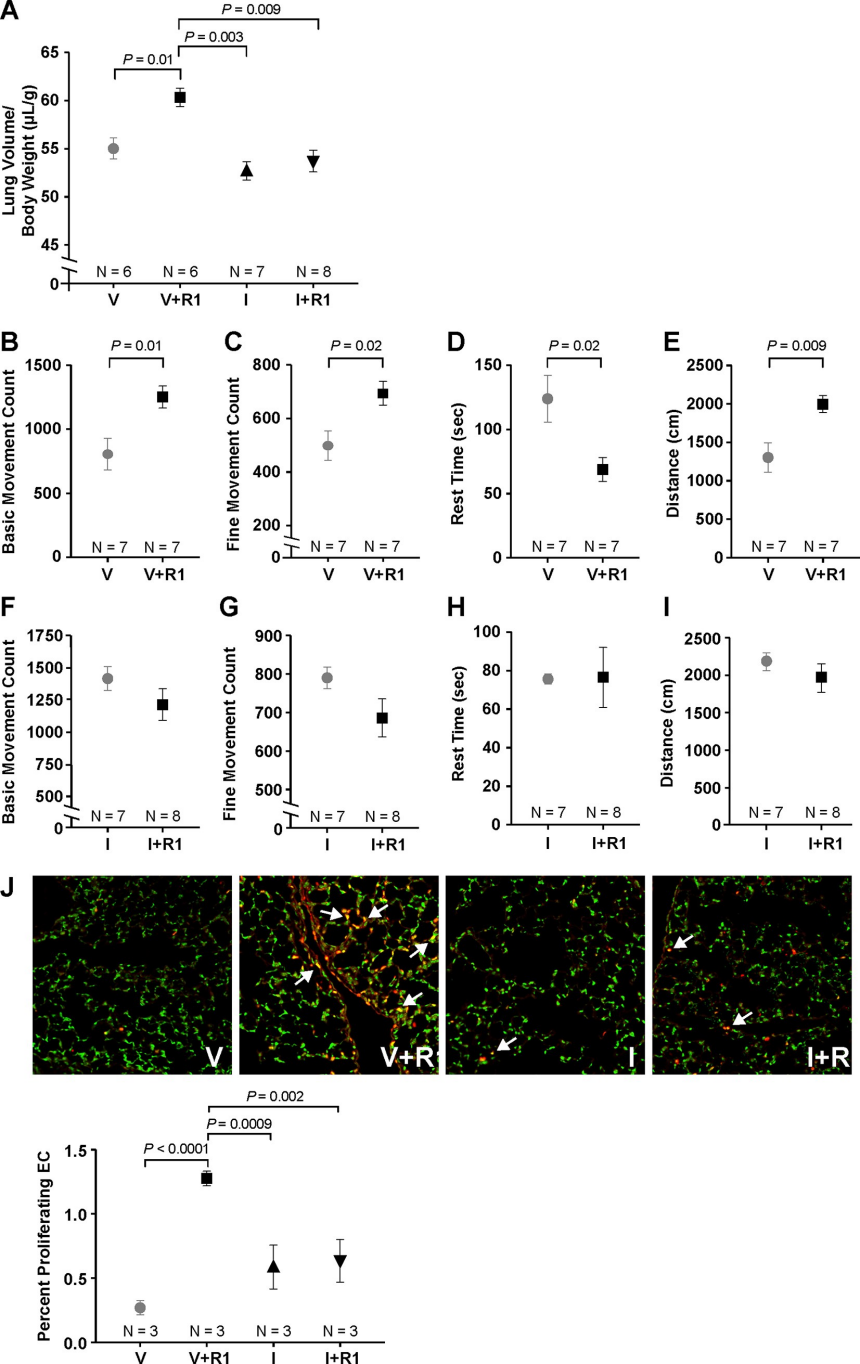
HIF2α neutralization abolished the effect of VEGFR1 on CLG. VEGFR1 treatment (F) again increased lung volume compared to the vehicle control group (V) (A). The administration of PT-2385, a HIF2α inhibitor, had no effect on lung volume without (I) or with (I+R1) VEGFR1. Compared to the V group, mice in the F group demonstrated increased physical activity as evidenced by increased basic and fine movement count (B and C), decreased rest time (D), and increase walking distance (E). These differences disappeared between the I and I+R1 groups (F-I). On IHC, there was an increased number of proliferating endothelial cells (white arrows) in the F group compared to the remaining 3 groups (J). There was no difference in endothelial proliferation between the I and I+R1 groups. Data are expressed as mean ± standard error (SE).

In order to confirm the role of HIF2α in mediating the effect of VEGFR1, we next investigated how HIF2α inhibition would alter CLG. PT-2385, a small-molecule inhibitor of HIF2α was administered via orogastric gavage every 12 hours. Compared to mice that received only vehicle gavage (V), the addition of VEGFR1 (V+R1) again improved lung volume (60.3 ± 1.1 vs 55.0 ± 1.1 μL/g, *P* = 0.01) (Fig 4A). In the presence of PT-2385, no difference in lung volume was observed with (I+R1) or without (I) VEGFR1.

Furthermore, physical activity was used as a surrogate for pulmonary function. Compared to the V group, mice in the V+R1 group displayed increased basic and fine movement count (*P* = 0.04 and 0.03, respectively), decreased rest time (*P* = 0.04), and increased walking distance (*P* = 0.03) (Fig 4B–4E). However, no difference in movement count, rest time, or walking distance was observed between the I and I+R1 groups (Fig 4F–4I).

On IHC, compared to the V group, the V+R1 group displayed increased endothelial proliferation (1.27 ± 0.07 vs 0.27 ± 0.07%, *P* < 0.0001) (Fig 4J). This result was consistent with previous findings of increased local expression of angiogenic factors on immunoblot and qPCR. In a similar fashion to lung volume measurement, the improvement in endothelial proliferation with VEGFR1 was neutralized with the addition of PT-2385 (Fig 4J).

Overall, these results again confirmed the positive effect of VEGFR1 at 20 μg/kg on CLG after left PNX. Furthermore, HIF2α plays a critical role in this process as its inhibition neutralized the improvement in lung volume, physical activity, and pulmonary angiogenesis seen with VEGFR1 treatment.

## Discussion

In this study, we demonstrated that the administration of VEGFR1-Fc, a VEGF inhibitor, at the dose of 20 μg/kg significantly improved lung volume, surfactant production, angiogenesis, and mechanical properties after left PNX. These results were likely mediated through stimulation of the hypoxia signaling pathway, as inhibition of HIF2α via a small-molecule inhibitor neutralized the effect of VEGFR1 on CLG.

VEGFR1 is a high-affinity receptor for VEGF and a well-known anti-angiogenic factor, whose applications range from anti-tumor to age-related macular degeneration therapy [12]. Paradoxically, VEGFR1-Fc administration resulted in an inverted U-shaped dose response, where CLG peaked at 20 μg/kg. We speculate that the lower dose of 5 μg/kg resulted in impaired CLG via VEGF inhibition while the optimal dose of 20 μg/kg activated a compensatory signaling pathway that counteracted the anti-angiogenic response. The higher doses of 60 and 180 μg/kg probably overwhelmed this compensatory signal, again resulting in decrement of lung growth. The response associated with these higher doses is probably similar to that seen in the adenoviral delivery method [19].

The increase in expression of VEGF, VEGFR2, and a number of other angiogenic factors confirmed our hypothesis that there exists a compensatory response which paradoxically stimulates angiogenesis in the face of VEGF inhibition. Since VEGF expression is under the control of HIF and HIF expression has been shown to upregulate with the administration of VEGF-Trap, a VEGFR1-VEGFR2 fusion protein, in the ovary [20], we next hypothesized that VEGFR1 treatment stimulated CLG by activating the hypoxic response. It is notable that of the two isoforms of HIF, only HIF2α showed upregulation after VEGFR1 administration. This result is consistent with other studies that demonstrate HIF2α, rather than HIF1α, is the major driver of lung growth and maturation [21–23]. Since post-pneumonectomy lung growth simulates the late alveolar stage of lung development [24], it is not surprising that VEGFR1 enhanced CLG in this study by specifically upregulating HIF2α. It should also be noted that this process was quite specific as the increase in VEGF expression was not seen in non-regenerating organs, such as the liver and the kidney. The increase in plasma VEGF concentration was possibly a result of increased local production of VEGF in the lung as a result of VEGFR1 treatment.

In this study, we demonstrated that the HIF2α-VEGF axis takes place in AEC type II, which is consistent with the published literature [25,26]. Activation of HIF2α-VEGF in turn elicited a series of responses that were essential to lung growth and maturation. The upregulation of EGF is notable as it is an important epithelial cell mitogen, whose levels have been shown to increase with exogenous VEGF administration [11]. This probably played a role in promoting lung maturation, as evidenced by increased production of surfactant proteins B and C. Given that AECII is also responsible for surfactant synthesis, it is therefore not surprising that both hypoxia and VEGF have been shown to be important promoters of SP-C production [27,28]. Taken together, VEGFR1 treatment stimulated the hypoxic response and enhanced CLG by stimulating both angiogenesis and mechanisms that affect lung maturation.

Therefore, VEGFR1 therapy could find clinical applications in the treatment of developmental lung diseases, such as CDH and BPD. Analyses of post-mortem lung tissues from children succumbing to CDH, a form of hypoplastic lung disease, revealed dysregulation of the HIF2α-VEGF axis in the alveolar stage of lung growth [29]. In an animal model of BPD, exposure to high levels of oxygen downregulated the expression of HIF and angiogenic factors [30]. Given the central role of HIF-VEGF in the pathogenesis of these diseases, targeting this pathway with VEGFR1 could represent a new therapeutic strategy for the treatment of these highly morbid and costly neonatal conditions [31,32].

Although this study focused exclusively on the process of CLG, VEGFR1 administration could induce certain systemic side effects. Most notably, VEGFR1 is implicated in the pathogenesis of preeclampsia [33], which would limit its applicability as a prenatal therapy. Furthermore, this study was also limited by its superficial investigation of other aspects of the HIF pathways. Our study placed the focus on angiogenesis, specifically VEGF, due to its critical role in lung growth and development. However, it is possible, if not likely, that other downstream effects of HIF activation, such as chemotaxis and vasodilation, can play a major supporting role in this process.

## Supporting information

S1 File Original data from all experiments. (XLSX)

## References

[1] Ferrara N, Gerber H-P, LeCouter J. The biology of VEGF and its receptors. Nat Med. 2003;9(6):669–76. 10.1038/nm0603-669 12778165

[2] Fong G-H, Rossant J, Gertsenstein M, Breitman ML. Role of the Flt-1 receptor tyrosine kinase in regulating the assembly of vascular endothelium. Nature. 1995 7 6;376(6535):66–70. 10.1038/376066a0 7596436

[3] Healy AM, Morgenthau L, Zhu X, Farber HW, Cardoso W V. VEGF is deposited in the subepithelial matrix at the leading edge of branching airways and stimulates neovascularization in the murine embryonic lung. Dev Dyn. 2000;219(3):341–52. 10.1002/1097-0177(2000)9999:9999<::AID-DVDY1061>3.0.CO;2-M 11066091

[4] Acarregui MJ, Penisten ST, Goss KL, Ramirez K, Snyder JM. Vascular Endothelial Growth Factor Gene Expression in Human Fetal Lung *In Vitro*. Am J Respir Cell Mol Biol. 1999 1;20(1):14–23. 10.1165/ajrcmb.20.1.3251 9870913

[5] Brown KR, England KM, Goss KL, Snyder JM, Acarregui MJ. VEGF induces airway epithelial cell proliferation in human fetal lung in vitro. Am J Physiol Lung Cell Mol Physiol. 2001 10;281(4):L1001–10. 10.1152/ajplung.2001.281.4.L1001 11557604

[6] Muehlethaler V, Kunig AM, Seedorf G, Balasubramaniam V, Abman SH. Impaired VEGF and nitric oxide signaling after nitrofen exposure in rat fetal lung explants. 2008;294(1):110–20. 10.1152/ajplung.00407.2007 17993583

[7] Chang R, Andreoli S, Ng Y-S, Truong T, Smith SR, Wilson J, et al VEGF expression is downregulated in nitrofen-induced congenital diaphragmatic hernia. J Pediatr Surg. 2004;39(6):825–8. 15185205 10.1016/j.jpedsurg.2004.02.015

[8] Thébaud B, Ladha F, Michelakis ED, Sawicka M, Thurston G, Eaton F, et al Vascular endothelial growth factor gene therapy increases survival, promotes lung angiogenesis, and prevents alveolar damage in hyperoxia-induced lung injury: evidence that angiogenesis participates in alveolarization. Circulation. 2005 10;112(16):2477–86. 10.1161/CIRCULATIONAHA.105.541524 16230500

[9] Kunig AM, Balasubramaniam V, Markham NE, Morgan D, Montgomery G, Grover TR, et al Recombinant human VEGF treatment enhances alveolarization after hyperoxic lung injury in neonatal rats. Am J Physiol Cell Mol Physiol. 2005 10;289(4):L529–35. 10.1152/ajplung.00336.2004 15908474

[10] Ding B-S, Nolan DJ, Guo P, Babazadeh AO, Cao Z, Rosenwaks Z, et al Endothelial-derived angiocrine signals induce and sustain regenerative lung alveolarization. Cell. 2011 10 28;147(3):539–53. 10.1016/j.cell.2011.10.003 22036563 PMC3228268

[11] Dao DT, Nandivada P, Vuong JT, Anez-Bustillos L, Pan A, Kishikawa H, et al Vascular endothelial growth factor accelerates compensatory lung growth by increasing the alveolar units. Pediatr Res. 2018 4 11;83(6):1182–9. 10.1038/pr.2018.41 29638228 PMC6019135

[12] Shibuya M. Vascular endothelial growth factor and its receptor system: physiological functions in angiogenesis and pathological roles in various diseases. J Biochem. 2013 1 1;153(1):13–9. 10.1093/jb/mvs136 23172303 PMC3528006

[13] Sakurai MK, Greene AK, Wilson J, Fauza D, Puder M. Pneumonectomy in the Mouse: Technique and Perioperative Management. J Investig Surg. 2005 1 9;18(4):201–5. 16126631 10.1080/08941930591004485

[14] Scherle W. A simple method for volumetry of organs in quantitative stereology. Mikroskopie. 1970 6;26(1):57–60. 5530651

[15] Livak KJ, Schmittgen TD. Analysis of relative gene expression data using real-time quantitative PCR and the 2^(-ΔΔCT) method. Methods. 2001;25(4):402–8. 10.1006/meth.2001.1262 11846609

[16] Wallace EM, Rizzi JP, Han G, Wehn PM, Cao Z, Du X, et al A Small-Molecule Antagonist of HIF2α Is Efficacious in Preclinical Models of Renal Cell Carcinoma. Cancer Res. 2016 9 15;76(18):5491–500. 10.1158/0008-5472.CAN-16-0473 27635045

[17] Mohamed AA, Tan S-H, Mikhalkevich N, Ponniah S, Vasioukhin V, Bieberich CJ, et al Ets family protein, erg expression in developing and adult mouse tissues by a highly specific monoclonal antibody. J Cancer. 2010 10 25;1:197–208. 21060730 10.7150/jca.1.197 PMC2974237

[18] Sakurai MK, Lee S, Arsenault DA, Nose V, Wilson JM, Heymach J V, et al Vascular endothelial growth factor accelerates compensatory lung growth after unilateral pneumonectomy. Am J Physiol Lung Cell Mol Physiol. 2007;292(3):L742–7. 10.1152/ajplung.00064.2006 17122356

[19] Panigrahy D, Kalish BT, Huang S, Bielenberg DR, Le HD, Yang J, et al Epoxyeicosanoids promote organ and tissue regeneration. Proc Natl Acad Sci. 2013 8 13;110(33):13528–33. 10.1073/pnas.1311565110 23898174 PMC3746918

[20] Duncan WC, van den Driesche S, Fraser HM. Inhibition of Vascular Endothelial Growth Factor in the Primate Ovary Up-Regulates Hypoxia-Inducible Factor-1α in the Follicle and Corpus Luteum. Endocrinology. 2008 7;149(7):3313–20. 10.1210/en.2007-1649 18388198

[21] Compernolle V, Brusselmans K, Acker T, Hoet P, Tjwa M, Beck H, et al Loss of HIF-2α and inhibition of VEGF impair fetal lung maturation, whereas treatment with VEGF prevents fatal respiratory distress in premature mice. Nat Med. 2002 6;8(7):702 10.1038/nm721 12053176

[22] Rajatapiti P, van der Horst IWJM, de Rooij JD, Tran MGB, Maxwell PH, Tibboel D, et al Expression of hypoxia-inducible factors in normal human lung development. Pediatr Dev Pathol. 2008 5;11(3):193–9. 10.2350/07-04-0257.1 17990921

[23] Jiang X, Tian W, Tu AB, Pasupneti S, Shuffle E, Dahms P, et al Endothelial HIF-2α is Required for the Maintenance of Airway Microvasculature. Circulation. 2018;[Epub ahead of print]. 10.1161/CIRCULATIONAHA.118.036157 PMC6340714 30586708

[24] Kho AT, Liu K, Visner G, Martin T, Boudreault F. Identification of dedifferentiation and redevelopment phases during postpneumonectomy lung growth. Am J Physiol Lung Cell Mol Physiol. 2013 10 15;305(8):L542–54. 10.1152/ajplung.00403.2012 23997171 PMC3798774

[25] Woik N, Kroll J. Regulation of lung development and regeneration by the vascular system. Cell Mol Life Sci. 2015 7 19;72(14):2709–18. 10.1007/s00018-015-1907-1 25894695 PMC11113134

[26] Mura M, Binnie M, Han B, Li C, Andrade CF, Shiozaki A, et al Functions of Type II Pneumocyte-Derived Vascular Endothelial Growth Factor in Alveolar Structure, Acute Inflammation, and Vascular Permeability. Am J Pathol. 2010 4;176(4):1725–34. 10.2353/ajpath.2010.090209 20167862 PMC2843464

[27] Shimoda LA, Semenza GL. HIF and the lung: role of hypoxia-inducible factors in pulmonary development and disease. Am J Respir Crit Care Med. 2011 1 15;183(2):152–6. 10.1164/rccm.201009-1393PP 21242594 PMC3159088

[28] Del Moral P-M, Sala FG, Tefft D, Shi W, Keshet E, Bellusci S, et al VEGF-A signaling through Flk-1 is a critical facilitator of early embryonic lung epithelial to endothelial crosstalk and branching morphogenesis. Dev Biol. 2006 2 1;290(1):177–88. 10.1016/j.ydbio.2005.11.022 16375885

[29] van der Horst IWJM, Rajatapiti P, van der Voorn P, van Nederveen FH, Tibboel D, Rottier R, et al Expression of hypoxia-inducible factors, regulators, and target genes in congenital diaphragmatic hernia patients. Pediatr Dev Pathol. 1;14(5):384–90. 10.2350/09-09-0705-OA.1 21671771

[30] Elberson VD, Nielsen LC, Wang H, Kumar HSV. Effects of intermittent hypoxia and hyperoxia on angiogenesis and lung development in newborn mice. J Neonatal Perinatal Med. 2016 1 14;8(4):313–22. 10.3233/NPM-15814134 26836820

[31] Principi N, Di Pietro GM, Esposito S. Bronchopulmonary dysplasia: clinical aspects and preventive and therapeutic strategies. J Transl Med. 2018 12 20;16(1):36 10.1186/s12967-018-1417-7 29463286 PMC5819643

[32] Morini F, Valfrè L, Bagolan P. Long-term morbidity of congenital diaphragmatic hernia: A plea for standardization. Semin Pediatr Surg. 2017 10;26(5):301–10. 10.1053/j.sempedsurg.2017.09.002 29110826

[33] Herraiz I, Llurba E, Verlohren S, Galindo A, Spanish Group for the Study of Angiogenic Markers in Preeclampsia. Update on the Diagnosis and Prognosis of Preeclampsia with the Aid of the sFlt-1/ PlGF Ratio in Singleton Pregnancies. Fetal Diagn Ther. 2018;43(2):81–9. 10.1159/000477903 28719896

